# Healthcare Providers’ Perspectives Regarding Barriers and Facilitators to Former Pap/VIA-Based Screen-and-Treat Program in Iquitos, Peru

**DOI:** 10.1007/s43477-025-00164-8

**Published:** 2025-05-02

**Authors:** Lauren Nussbaum, Joanna Brown, Graciela Meza Sánchez, Sandra Soto, Magdalena Jurczuk, Javier Vásquez Vásquez, Henrry Daza Grandez, Lita E. Carrillo Jara, Renso López Liñán, Patti E. Gravitt, Valerie A. Paz‑Soldán

**Affiliations:** 1 Celia Scott Weatherhead School of Public Health & Tropical Medicine at Tulane University, New Orleans, USA; 2 Asociación Benéfica PRISMA, Lima, Peru; 3 Facultad de Medicina Humana, Universidad Nacional de la Amazonía Peruana, Iquitos, Peru; 4 Gerencia Regional de Salud de Loreto, Iquitos, Peru; 5 Horizon Praxis, Baltimore, MD USA

**Keywords:** Inner setting, Cervical cancer, Human papillomavirus, Peru, CFIR

## Abstract

Longstanding structural barriers to Pap smear-based cervical cancer screening and treatment have existed in Peru for decades. The objective of this study was to understand healthcare providers’ perspectives regarding the facilitators of and barriers to the now former Pap/Visual Inspection with Acetic Acid-based cervical cancer prevention program in Iquitos, Peru, to inform the transition to the human papillomavirus (HPV) molecular testing-based screen-and-treat intervention to increase screening and completion of care. We used constructs from the Consolidated Framework for Implementation Research’s Inner Setting domain to understand the strengths and failures of the former system and leverage this knowledge to enhance the new HPV-based intervention’s implementation. We conducted 19 semi-structured interviews with health professionals (12 nurse-midwives, 4 doctors, and 3 laboratory technicians) who administered the former Pap smear-based cervical cancer early detection and treatment program. Providers identified information gaps between the primary level of care, where cervical cancer screening occurs, and the hospital level of care, where diagnosis and treatment occurs. These gaps, caused in part by fragmented and overlapping data systems that do not connect with one another, as well as by healthcare professionals tending to concentrate solely on their own specific role, rather than recognizing the importance of all components working cohesively to facilitate completion of the continuum of care, resulted in the loss of patients between levels of care. Participants also noted a lack of trained personnel and basic materials. Some providers found their way around these gaps by facilitating informal information exchanges among providers to ensure women were not lost to follow-up. Proyecto Precáncer leveraged these findings by collaborating with stakeholders to map the former system, reach stakeholder consensus on system inefficiencies, and design an intervention that improved system efficiencies through a patient-centered approach.

**Supplementary Information:**

The online version contains supplementary material available at 10.1007/s43477-025-00164-8.

## Assessing Inner Setting in Loreto, Peru, Prior to Transition from Pap Smear to HPV Screen-and-Treat

Cervical cancer is one of the leading causes of female cancer-related mortality in Peru (World Health Organization, 2020), and the Loreto region of Peru experiences a mortality rate 2.6 times higher than the national average (Piñeros et al., 2017). This study is part of Proyecto Precáncer, an implementation science project comprised of public health authorities, providers, and researchers that is implementing a new cervical cancer early detection and treatment (EDT) program at the primary level of care to increase screening and completion of care (Gilman et al., 2024; Gravitt et al., 2020). This program, which launched in Iquitos, Loreto’s capital city, in July 2019, (1) transitioned from Pap smear and visual inspection with acetic acid (VIA) screening to HPV screening with clinician and self-sampling options, and (2) consisted of a visual assessment for treatment (VAT) for all women who tested positive for HPV, with thermal ablation with thermocoagulation at the primary level for those eligible (no acetowhite lesions or lesions covering less than 75% of transformation zone), and referral to a specialist at the local hospital for those ineligible for thermal ablation. Within 6 months of implementation, the new HPV-based EDT program surpassed the WHO elimination target of screening 70% of age-eligible women, reaching a peak of 297.7 tests per month (128% of the screening goal) and reducing the loss to follow-up rates from 66.6% (402/604) with VIA to 30.2% (52/172) with HPV screening and treatment (Gravitt et al., 2020; WHO, 2014).

Implementation science, which promotes the uptake of evidence-based practices, plays a crucial role in streamlining the implementation and scale up of new interventions (Bauer et al., 2015), including HPV screening and treatment interventions (Paolino et al., 2023). By focusing on key factors, including the acceptability, appropriateness, feasibility, and fidelity to the intervention design, implementation research can optimize the future implementation processes so that innovations are effectively and efficiently delivered to those who most need them (Proctor et al., 2011).

Using the Consolidated Framework for Implementation Research (CFIR) (Damschroder et al., 2022), the objective of this study was to understand the perspectives of the healthcare providers regarding the facilitators of and barriers to the former Pap smear- and VIA-based cervical cancer EDT program prior to implementation of the intervention. The few studies that have used CFIR at the pre-implementation stage have been useful in revealing factors that may impact an intervention’s success (Fokuo et al., 2022; Taylor et al., 2020). We explored the opinions of those who would implement the HPV-based intervention at the patient care level to understand the organizational capabilities, tasks, and processes necessary to design and implement a contextually appropriate and sustainable HPV-based intervention in Iquitos.

## Methods

### Study Setting

Loreto is the largest state of Peru and is located in the northern Amazon River basin. Iquitos (estimated population 442,000 (CPI Research, 2023)), is the largest city in the world that is not accessible by roads; due to the dense surrounding rainforest, it can be reached only by air or boat. Most communities around Iquitos are connected by rivers.

This pre-implementation assessment took place in the *Micro-Red Iquitos Sur* health network, which consists of 17 health facilities with a range of services; some with only one nurse-midwife (*obstetra*) and others with multiple doctors, midwives, technicians, and small laboratories. There are two hospitals where women can be referred for tertiary care. The *Micro-Red Iquitos Sur* serves a population of 20,000 women between 30 and 49 years who are eligible for the screen-and-treat approach using HPV testing and ablative therapy. Most (67%) of the population of Loreto is covered by the *Seguro Integral de Salud*, a public health insurance program that provides freely available cervical cancer screening and care at the health facilities in the *Micro-Red Iquitos Sur*.

### Participant Selection, Procedures, and Data Collection

During Proyecto Precáncer’s time in the *Micro-Red Iquitos Sur*, we gathered data to aid in the implementation of the HPV screen-and-treat program using the INSPIRE model, which has been described in detail elsewhere (Gilman et al., 2024; Gravitt et al., 2020). In short, this model consists of four phases:

- Phase 1: Understanding the Pap smear- and VIA-based cervical cancer EDT program.
- Phase 2: Identifying opportunities to improve the EDT program.
- Phase 3: Strategically implementing a new, internally developed EDT program (the HPV screen-and-treat program was the result of this co-design process).
- Phase 4: Continuously monitor and evaluate the new EDT program to learn and adapt.

As part of INSPIRE Phase 1, we conducted this pre-implementation assessment through 19 semi-structured interviews with health professionals. We used purposive and snowball sampling strategies, initially recruiting healthcare workers that were integral to the former Pap smear- and VIA-based cervical cancer EDT program (i.e., the head of the gynecology department at the local hospital, the laboratory technician who analyzed Pap smears from the health centers), and then asking those participants for suggestions of other healthcare providers we should approach. All participants worked in the *Micro-Red Iquitos Sur* primary health centers (seven of 17 facilities) or public hospitals (two of two facilities). Our research team contacted participants about the interviews at the health facility where they worked or over the phone, and all participants provided written, informed consent prior to each interview. Between March and October 2017, three members of the research team interviewed 11 hospital-level and 8 primary-level healthcare staff: 12 midwives, 4 doctors, and 3 laboratory technicians. Interviews were primarily conducted by VPS, the study MPI who is a Peruvian–American social scientist with ample qualitative research experience and based in Peru; some interviews with midwives were conducted by JM, a local research assistant with a college degree in biology, and MJ, an American MPH student, fluent in Spanish, who lived in Iquitos 18 months). These 30–60 min in-person interviews were conducted in Spanish with participants in a private area of the health facility where they worked. The researchers relied on an interview guide that was focused on participants’ understanding of the Pap smear- and VIA-based cervical cancer EDT program, including their understanding of the flow of information through the system (see Additional File 1 for Interview Guide). All interviews were recorded and transcribed. The transcripts were not shared with the participants. Recruitment ceased upon achieving thematic saturation across stakeholder levels regarding the barriers to and facilitators of the former Pap/VIA-based system. All of the participants were later involved in the group model building process that designed the HPV-based intervention that was implemented in Iquitos in 2019.

Ethics approval for this study was obtained from the local institutional review boards at Asociación Benéfica PRISMA, Hospital Regional Loreto, and Hospital Apoyo Iquitos, as well as by the institutional review boards at the multiple-PIs’ institutions, Tulane University School of Public Health and Tropical Medicine and the University of Maryland School of Medicine. As approved by these institutional review board committees, all participants provided written informed consent to participate.

### Data Analysis

The updated CFIR, which can be applied to identify and assess contextual factors that can facilitate or hinder implementation of novel innovations, is made up of five domains: Innovation, Outer Setting, Inner Setting, Individuals, and Implementation (Damschroder et al., 2022). We analyzed participants’ transcripts using deductive codes based on the CFIR’s five domains and their associated constructs and subconstructs. Using Dedoose (Dedoose Version 8.0.35, 2018), two researchers (JB and SS, both Peruvian psychologists with qualitative research experience) read and coded the 19 transcripts and met to discuss discrepancies in coding and adjust the codebook as needed. One researcher (SS) then coded all remaining transcripts, and the research team met to discuss themes that emerged from the data. The great majority of topics that emerged, in part due to our interest in hearing providers’ perspectives on current system practices and potential readiness for change, fell within the Inner Setting domain; hence, this analysis focuses on this domain. Surveys and focus groups with women (Individuals domain) and interviews with federal policy makers (Outer Setting domain) are reported in other manuscripts (Barrett et al., 2020; Morse et al., 2023, 2024;) or are in progress.

## Results

The Inner Setting domain—the focus of this analysis—represents the site-specific organizational culture and structural context for intervention implementation (Fernandez et al., 2018; Fokuo et al., 2022; Walker et al., 2019). We do not address two Culture Setting subconstructs from this domain (Human Equality Centeredness and Recipient Centeredness) because those two subconstructs did not emerge from the interview responses. Further, we did not include Constructs F-K because they are specific to the implementation and/or delivery of the innovation (Damschroder et al., 2022), and thus were not relevant to this pre-implementation context. The constructs and subconstructs used, along with their definitions, are shown in Table 1.

**Table 1:** Coding tree of inner setting constructs and subconstructs used

Construct	Subconstruct	Definition
Structural characteristics		Infrastructure components support functional performance of the Inner Setting
	Physical infrastructure	Layout and configuration of space and other tangible material features support functional performance of the Inner Setting
	Information technology infrastructure	Technological systems for tele-communication, electronic documentation, and data storage, management, reporting, and analysis support functional performance of the Inner Setting
	Work infrastructure	Organization of tasks and responsibilities within and between individuals and teams, and general staffing levels, support functional performance of the Inner Setting
Relational connections		There are high quality formal and informal relationships, networks, and teams within and across Inner Setting boundaries (e.g., structural, professional)
Communications		There are high quality formal and informal information sharing practices within and across Inner Setting boundaries (e.g., structural, professional)
Culture setting		There are shared values, beliefs, and norms across the Inner Setting. Use this construct to capture themes related to Culture that are not included in the subconstructs below
	Deliverer-centeredness	There are shared values, beliefs, and norms around caring, supporting, and addressing the needs and welfare of deliverers
	Learning-centeredness	There are shared values, beliefs, and norms around psychological safety, continual improvement, and using data to inform practice
Tension for change		The current system is intolerable and needs to change

### Structural Characteristics: Physical Infrastructure

Participants noted that they lacked materials and equipment that were necessary to implement the EDT program. While doctors noted that they needed equipment to perform treatment, such as biopsy forceps and cryotherapy machines, midwives noted that they often needed various simple supplies, such as examination beds, examination lamps, swabs, vinegar, and specula. The lack of basic supplies to facilitate care was even more apparent at the primary level facilities outside the city center, which can lack consistent running water, electricity, and phone lines. When the healthcare facilities ran out of their own supplies, it became the patients’ responsibility to provide equipment such as gloves, pain medication, swabs, or even specula.

Sometimes the vagina is wider, longer, and sometimes the speculum cannot reach it, and sometimes we do not have the necessary supplies. They only give us medium size speculums, which might be a bit uncomfortable at the time of performing the pap smear… I have to call the technical staff so that they can give me light and I can reach the cervix. (Primary-level midwife)

They should stock the facility well with biopsy forceps, with everything, because it is not well stocked. We don’t have those things. In other words, if I want to perform a biopsy, I have to stop and tell the patient that I have to coordinate with the laboratory. Sometimes, the lab technician, because he’s a nice guy, will lend me forceps, but that’s something that we don’t have. Thanks to our friendship I can access those things, but we don’t have them. (Hospital-level doctor)

### Structural Characteristics: Information Technology Infrastructure

Participants identified a fragmented health records system as the main barrier in this subconstruct. Many primary care health centers used a paper-based system for recording screenings, in part due to a lack of computers and/or internet at the facilities. In fact, in addition to filling out numerous forms that take up a lot of their time, most midwives keep their own records in notebooks to keep track of those with whom they must follow up. Paper forms they fill out are then sent to a single facility, where the files are entered in an electronic system. However, midwives reported feeling that the electronic system often does not reflect the information sent about the screenings performed, resulting in primary care providers being reprimanded for not meeting screening goals, causing demoralization: “We worked hard last year and the system did not work. This year we are already reviewing the information, we have to show all our work, because it was not fair last year.” (Primary-level midwife).

For primary care centers that did have electronic data systems in place, midwives noted that the systems were difficult to use because they were constantly changing. Further, these electronic and paper-based systems were fragmented, overlapping, and not user-friendly: “They always offer training on how to fill out the [electronic system], it is always changing. I, for example, have used the [electronic system] from 2012 to 2013, I think, then it changed in 2015.” (Primary-level midwife).

These documents are the advice and counseling that we give to each patient. And this is our Pap smear registry. Now we have another registry, the VIA registry, with reports and registries for that. In terms of a registry that shows who is due for a Pap smear this month, who we need to follow up with, we don’t have that specific registry. What we do is we perform follow-up according to the clinical histories. We can see, “last year my colleague did a Pap smear for this patient, but not for that patient, and here is the result.” On their family planning card, there is a box for Pap smears, if they had one, when was the last time they had one, etc. But a registry specifically for Pap smears that contains all the patients, and when they are due for the next Pap smear, we don’t have that. (Primary-level midwife)

### Structural Characteristics: Work Infrastructure

Providers identified the lack of patient information data flow between providers within the system as a principal barrier. Although the referral system is designed for a referral to a higher level of care, and then a counter-referral back to the primary level, that does not occur.

Once she is referred [to the hospital for treatment], I am not informed about what has happened, if she was treated, if she came out positive, or not.... She is now a hospital patient, but I have to follow up with her to see what is happening with her. (Primary-level midwife)

Midwives at the primary level feel responsible for any patient follow-up required, yet they have to either call the hospital midwife for information about their patients or follow up with their patients (and patients are not always able to explain what happened in their follow-up). Likewise, gynecologists who refer patients to the oncology department of their same hospital for further analysis, or those who refer patients with a cervical cancer diagnosis to Lima for treatment, do not know what happens to their patients.

I don’t know how oncology works, I imagine it works fine, but if I refer a patient to oncology, that’s not great because then we are passive entities. I send the Pap smear results but I don’t know if the patient will go to oncology…. When I refer her for a biopsy, I don’t perform that biopsy, another doctor does. I don’t know if the patient will go or not, and I lose her. (Hospital-level doctor)

Providers are not formally allocated time and resources to follow up with patients whom they have referred for further screening or treatment at the different system levels. Follow-up is performed at the provider’s discretion; some providers do what they can to fill those gaps so that women are not lost.

This is a tertiary level hospital. We are not a health center. The health centers do the follow-up visits, all of them. We give them a date for their -up visit [at the health centers], 15 days, 1 month, 3 months. But if the patient does not attend that appointment, that is her problem. We do not do follow up! (Hospital-level doctor)

Theoretically, everything, VIA and Pap smears, should be performed at the primary level of care. Here at the tertiary level of care, per Ministry of Health norms, Pap smears should not be performed here. That’s the ideal, but here we are performing Pap smears so that we don’t lose patients. (Hospital-level doctor)

But patients know that Saturdays are the day for Pap smears. In other words, just Saturdays are for Pap smears and VIA, but if a patient comes for those services during the week, I will do it then, I don’t wait for Saturday…. Perhaps she’s there during the week because that’s when she has time. I try to accommodate the patients that work, because most people here work, whether in their homes, or outside of the city. (Primary-level midwife)

Providers noted a lack of personnel outside of Lima, and specifically in Loreto, who are trained to read laboratory results, provide treatment, or perform needed follow-up care. For example, one of the hospital’s sole pathologists can read approximately 80 Pap smear samples per day, but the hospital takes 120–130 Pap smear samples every day, so the backlog keeps accumulating. In addition, there is often only a single doctor in the hospital who is trained to perform cervical cancer treatment procedures, such as conization or loop electrosurgical excision procedures, and so when that doctor is on leave, retires, or leaves his position at the hospital, that procedure cannot be performed until another doctor receives the requisite training. Due to limited budgets, providers are unable to devote time to follow up with patients once they have been referred elsewhere.

### Relational Connections

A key obstacle to high quality relationships that emerged was the bureaucratic, hierarchical organizational culture, where providers stick to their explicitly delineated tasks.

Interviewer:And when you send them to the National Cancer Institute [for treatment in Lima], is there anyone in charge of follow-up for these patients who were referred for treatment?

Participant: Follow-up, no, we don’t do that, it’s in our work plan to perform follow-up care, but we don’t have the personnel to do that follow-up.
Interviewer: You don’t do follow-up.
Participant: For patients treated in Lima, no. When do we see that patient? When she comes back. And how do we know when she has died? When someone comes to request her death certificate. There is a gaping hole. (Hospital-level midwife)
Interviewer: Who should be trained to perform cryotherapy, only doctors, or midwives too?
Participant: Don’t be foolish, just doctors! Midwifes do other things. I was in a meeting with midwives last week and it was noted that cryotherapy should be implemented at tertiary and quaternary level facilities. Stick to what you know, a midwife will want to perform up to chemotherapy, but they have their training, in their field. This is the fight they are in! Sometimes I receive prescriptions from midwives here, they should not be prescribing, but they do it anyway. They perform ultrasounds! And the Medical College [of Peru] is very clear that midwives should not be performing ultrasounds. Here I have midwives that are getting their specialty, but they are not allowed to do colposcopies here, and definitely not conization. They have their field of training.
Interviewer: But if they were properly trained, could they do it?
Participant: If the Medical College gives its authorization, they could, but I’m not going to break the rules! That’s not going to happen, at least not in my hospital. I don’t know if they would let that happen in the health centers, it would depend on the doctor there.
Interviewer: It’s a question worth asking.
Participant: You’d have to talk to the Medical College [professional society of medicine in Peru], the midwives want to, but they are not doctors. They say they could do it.... I could perform a cold cone biopsy, but I shouldn’t, that’s what the gynecologists are for! I think I would be capable of performing a cold cone biopsy, I can already do a Loop Electrosurgical Excision Procedure, I think I’m capable of doing a cold cone biopsy, but I won’t, I stick to what I know. (Hospital-level doctor)

### Communications

We already noted above the information gaps that exist across different levels of care, as well as the incomplete patient records and electronic data systems. However, providers found their way around these gaps by facilitating informal information exchanges among providers through phone calls and text messages (e.g., WhatsApp).

If I want to refer a patient for a gynecological consult, I pick up the phone and say, “You know what, I have a patient for you.” It’s immediate, so that we don’t lose the patient. Other providers call me when they have someone for me to see, I tell them, “Sure, have her come right now.” Because if we depend on the administrative side, going through the referral process, the patient will first have to make an appointment. (Hospital-level doctor)

To facilitate things a bit, we’ve created a WhatsApp group, so that my colleagues already know who is in charge [of cervical cancer] and that we work here, so when they have a referral, they call me and say, “I’m sending you a patient!” Sometimes the patient comes straight here with her referral. (Hospital-level midwife)

### Culture Setting: Deliverer- and Learning-Centeredness

Overwhelmingly, providers noted that a lack of shared norms regarding their professional development and quality control. Specifically, participants clamored for more frequent trainings that should contain consistent content and be accompanied by a system for quality control.

The trainings should be more frequent, because it doesn’t always stay in your head, and things are constantly changing. For example, regarding the light bulb, I didn’t know white light was better for VIA, and so after that training, I changed my light bulb from a yellow light to a white light, but then they told me no, that I should continue with the yellow light bulb. I responded, “But in a training in 2012 you told us to use white light,” but then in 2016, it was yellow light. But I wasn’t able to attend that later training, so I didn’t know that.... The trainings should be annual. (Primary-level midwife)

For me, the best thing that we could do is strengthen the basic skills regarding taking samples. Supervising, that would take 3 or 4 months. And then, after ensuring that everyone is performing well, to have an executive director that monitors the quality. You have to perform quality control using the reports that the system provides. The [2001] Bethesda System is a very good system that allows you to supervise the samples being taken. (Laboratory technician)

### Tension for Change

This is the only construct specific to the implementation and/or delivery of the innovation that we included because the need for innovation emerged organically from the interviews. Specifically, providers recognized that the fragmentation between screening and treatment created bottlenecks and gaps in timely treatment that then led to patients lost to follow-up. Some hypothesized that a superior data system would facilitate improvement. Others acknowledged the lack of personnel trained to read samples was causing delays, and suggested investing in this human resource and decentralizing it.

If we had [an adequate] registry, we would see where things get stuck: where there are delays, if there has been a delay in the referral, in the treatment. Well-managed statistics, or records in this case, would allow us to see the reality of the work we are doing. (Hospital-level doctor)

Here, there’s a large number of samples to be read, but only one person can read them… I am convinced—and I think we all have the same opinion—that by training more personnel and gathering them to set up the laboratory here, this whole problem would be solved. One, because we would no longer be sending samples to the Regional Hospital… we would do it here. (Hospital-level doctor)

Similarly, others suggested shifting treatment to the primary level of care would improve the program, but identified the policy barriers associated with that change, particularly Ministry of Health approval. Another popular idea was sending more specialists to Iquitos, particularly clinical and surgical oncologists, so that patients with more advanced lesions do not have to be sent to Lima for treatment. Finally, providers suggested creating a counter-referral system, so that primary level providers know what screening and/or treatment their patients had at the hospital level and the results of those procedures.

The primary level has to be well reinforced, because the primary level does not solve anything…. In the primary level there should be, for example, colposcopies, that’s important, a well-trained doctor, VIA for everyone, Pap smears for everyone, but that has to be a policy of the [regional health system], of the Ministry! (Hospital-level doctor)

## Discussion

This study used constructs and subconstructs from CFIR’s Inner Setting domain to identify, from the relevant healthcare providers’ perspectives, the barriers and facilitators associated with the former Pap smear- and VIA-based cervical cancer screening and treatment program in Iquitos, Peru. Reported challenges associated with the Pap/VIA-based screening program included fragmentation between screening, performed at the primary level, and treatment, performed at the hospital level; lack of a registry or data system for managing patients requiring follow-up; lack of communication between different health system levels, as well as a hierarchical dynamic between types of health facilities, types of health providers, and even between authorities from the national and regional and local levels.

The fragmented patient care was associated with the finding that healthcare professionals tended to concentrate solely on their own specific role, rather than recognizing the importance of all components working cohesively and understanding that all are part of a larger, interconnected system; all system parts must work together to ensure that early detection of a problem results in necessary management. Patients were lost to follow-up due to a lack of open communication and data-sharing—in part due to the lack of a registry or data system that allowed for efficient information sharing—between the midwives screening patients and the hospital doctors treating them, as well as the length of time between screening and treatment, a finding that has been previously documented in Peru (Paz-Soldán et al., 2012). Moreover, this problem is not unique to Peru; it has consistently been highlighted in the literature regarding implementation of screen-and-treat programs in resource-limited settings (Holme et al., 2017; Lott et al., 2021; Selmouni et al., 2022; Thomson et al., 2020). Proyecto Precáncer incorporated this feedback in two ways. First, during group model building workshops, we focused on the importance of the continuum of care, by using visual mental models to ensure all stakeholders understood that early detection is not useful unless followed by appropriate management and treatment. Second, the Proyecto Precáncer and collaborators tackled identified obstacles in the pre-cervical cancer continuum of care in the HPV-based intervention by shifting most HPV treatment to the primary level of care via use of ablative therapy, which was generally performed during the same visit as the VAT (Gilman et al., 2024). This approach meant that most women could receive treatment in the same primary level healthcare facility where they were initially screened, thus mitigating the issues with referrals, counter-referrals, and follow-up work that midwives had to perform to ensure that women who tested positive actually received follow-up care.

Further, participants expressed significant frustration with the former data system, which impeded their inability to track their patients as they moved across different levels of care. There were discussions about time spent filling out forms, the number of forms that they are expected to fill, and the lack of any useful feedback regarding the information that they are expected to record. Indeed, the literature has noted that electronic data systems must be consistently accessible to all involved providers and provide comprehensive and accurate data so that healthcare providers can identify patients who need treatment and/or follow-up care, including follow-up screenings (Holme et al., 2017; Morse et al., 2024; Thomson et al., 2020; Zhu et al., 2022). Proyecto Precáncer addressed this barrier in the intervention by facilitating the design of a hybrid paper-electronic data system, for which midwives could fill a triple-copy form that satisfied health system documentation needs and facilitated adequate patient tracking between levels of care (Gilman et al., 2024).

Organizational culture, specifically a hierarchical structure where norms and practices are mandated from above without input from those on the ground, was another theme that emerged in the interviews and has been identified in other studies as another barrier to successful EDT programs (Simwinga et al., 2024). In Peru, as in other contexts (Arrossi et al., 2021; Obol et al., 2020; Selmouni et al., 2022), most healthcare policies are developed at the national Ministry of Health—in the nation’s capital, a very different context, with limited attention or detail about potential site-specific adaptation that is needed for comprehensive implementation and/or scale-up. Proyecto Precáncer used this finding to tackle the hierarchical culture by continuously engaging stakeholders at all levels of the system to ensure wide buy-in, including at the national level (Gilman et al., 2024), which has been shown to lead to more successful and sustainable interventions (Fokuo et al., 2022; Lopez et al., 2024). Additionally, Proyecto Precáncer focused on identifying local champions who could lead working groups and decision-making as the new strategy was designed, hence ensuring local ownership so that sustainability was not dependent on an outside research team or the Ministry of Health.

Participants emphasized that existing resources—regarding personnel as well as equipment—were insufficient to support implementation of the former EDT program. Shifting from a Pap smear- and VIA-based screening program to an HPV-based program did not necessarily solve these problems; rather, both methods have their advantages and disadvantages (Holme et al., 2017; Lott et al., 2021). Specifically, an HPV-based program relieves some burden on the human capacity needed to analyze samples by shifting that work to a machine, and substitutes a subjective test with an objective one, which addresses VIA’s issues regarding quality control (Holme et al., 2017; Moucheraud et al., 2020; Poli et al., 2020; Selmouni et al., 2022). A VIA-based program is attractive due to its low costs because it does not require advanced technology (Lott et al., 2021; Paz-Soldán et al., 2012), while an HPV-based program requires an expensive machine to read the DNA samples, as well as specific cartridges and cervical swabs that have been difficult for Proyecto Precáncer to consistently procure. Therefore, challenges remain regarding access to needed materials for an HPV-based program. This reality reinforces the conclusion that the impact of introducing improved technologies in low-resource settings may be limited if the intervention is discrete, that is, not accompanied by broader efforts to strengthen the health system as a whole (Moucheraud et al., 2020). Additionally, regarding insufficient provider training and how this relates to moving to an HPV-based system, the Proyecto Precáncer met with local authorities to ensure that new trainings took place and health education materials were developed and distributed. We plan to develop strategies to address the need for health personnel training by exploring different opportunities for continuous education.

Some participants explained how, despite all these challenges, including those related to communication within the Inner Setting, they followed through for their patients by creating informal information channels among providers, tracking down patients to ensure they weren’t lost between the different levels of care, and even chipping in for transportation so patients could travel to Lima for treatment. An enthusiastic, committed work force is crucial to motivating women to get screened in the first place; finding leverage within the existing system to opportunistically identify patients who are already seeking healthcare is of utmost importance in a resource-limited setting (Selmouni et al., 2022).

This study had several limitations that should be considered. First, by using purposive sampling and focusing only on the perspectives of providers, findings in this manuscript provide only a partial perspective of the barriers to and facilitators of an HPV-based screening and treatment system and are not representative of other perspectives or regions of Peru (Moucheraud et al., 2020). Second, our sample size of healthcare providers was small, limiting the generalizability of our findings. However, these data were validated by these participants and other stakeholders through the system audits, process mapping, and group model building workshops conducted during INSPIRE Phases 1 and 2 (Gilman et al., 2024; Morse et al., 2024).

## Conclusion

This study used CFIR’s Inner Setting constructs to understand healthcare providers’ perspectives regarding the facilitators of and barriers to the success of the former Pap- and VIA-based cervical cancer EDT program to inform the design of and transition to the HPV-based screen-and-treat intervention in Iquitos, Peru. The former cervical cancer prevention system suffered from various challenges, including fragmentation and knowledge gaps between levels of care and along the detection-diagnosis-treatment continuum of care. Proyecto Precáncer leveraged these findings by collaborating with stakeholders to map the former system, reach a shared understanding of the system and its flaws, and agree on a task-shifting and locally co-designed intervention on the shared knowledge about evidence and options available to them. However, challenges remain regarding resources and sustainability related to HPV technology, including procurement. This research lays the foundation for future implementation science researchers to understand the organizational capabilities, tasks, and processes necessary to design a contextually appropriate and sustainable intervention in a resource-limited setting.

## Supplementary Information

Below is the link to the electronic supplementary material.

Supplementary file1 (PDF 174 KB)

## Data Availability

The dataset supporting the conclusions of this article are available upon reasonable request and with permission from the participants.
