# Supplementary material for: Healthcare Providers’ Perspectives Regarding Barriers and Facilitators to Former Pap/VIA-Based Screen-and-Treat Program in Iquitos, Peru
Source: Glob Implement Res Appl. 2025 May 2;5(3):407–17. doi: 10.1007/s43477-025-00164-8 (PMC12361280; doi:10.1007/s43477-025-00164-8)
Supplement: Supplementary file 1 — Supplementary file1 (PDF 174 KB) [file 43477_2025_164_MOESM1_ESM.pdf]

“Developing and improving strategies for cervical cancer prevention in Peru”

Guidelines for meetings and interviews with collaborators

At the beginning of each work meeting, participants will provide their informed consent. In case the activities are conducted in person, the informed consent will be written, and in case it is virtual or by telephone, the consent will be virtual and recorded.

a) Introduction
   a) Presentation and explanation of the meeting/interview objectives

We thank you in advance for your time and willingness to participate. The objective of this meeting/interview is to explore the experiences and perceptions of healthcare professionals and key stakeholders regarding the implementation of cervical cancer screening and treatment. We will also discuss molecular-based alternatives, their implementation and monitoring, as well as the training and resources required for this purpose.

There are no right or wrong questions or answers. We are only interested in hearing about your experiences and opinions.

b) Community: awareness and access

Discussion will focus on community-level knowledge of cervical cancer and screening, current efforts by public institutions to raise awareness about the disease, and how information, communication and access can be improved.

- What information, education and communication issues are the most important for us to address today? (Mapping options)

- Knowledge and attitudes regarding cervical cancer and screening:
    - When do women typically undergo screening? (e.g., when they identify symptoms, during regular screening as a routine practice, other check-ups, etc.)
    - What do women say about cervical cancer?
    - What are the common myths about Pap/VPH testing? What are the main reasons women do not get screened? What can be done to address this?

- Health campaigns:
    - What is being done to promote the need for Pap/VPH testing? What can be improved?
    - Have there been campaigns to raise awareness of the need for Pap/HPV testing? How effective have they been?
    - What factors contributed to the campaign? Were there any challenges?
    - What could be improved?
    - How did people react to these campaigns?

- Screening program penetration
    - Do you believe women who need access to cervical cancer screening are getting it? Why? How can it improve?
    - What is the most effective way to reach these women? Who should be involved? What would be the challenges?
    - If you could improve anything about cancer cervical services in your community, what would you improve?
    - Do you believe cervical cancer screening services and education services can be improved at the community level? What is your role in this process?
    - What is the role of DIRESA?

- What is the role of the municipality?

HPV vaccines

- What is your perception of coverage among young women?
- What barriers have you heard of regarding access to the vaccine?
- What could we do to improve HPV vaccination coverage among young women in this community?
- What could be your role in this process?

c) Screening techniques flowchart
- How is the implementation process for VIA, Pap, and HPV testing conducted here in Iquitos? What steps are involved? *The objective is to review each technique and its implementation process in detail.*
- What are the main issues related to these techniques? What are their advantages and disadvantages?

  Variables to consider (the goal is to understand these and other influencing factors)
  - Sensitivity (the effectiveness of the technique (lost cases, false positives, false negatives)
  - Cost (laboratory equipment, human resources, training, etc). How convenient/affordable is it in the Iquitos context?
  - Time (immediacy/delay of results)
  - Laboratory needs (infrastructure, equipment)
  - Logistical needs (e.g. does it require electricity? is sample transportation fast and effective?)
  - High medical knowledge requirement (How demanding is it to implement? What type of training does it require? For whom? By whom?)
  - Risks of overtreatment or undertreatment
  - How equitable is it? Would the general population have access? How easy or difficult would it be?
  - Feasibility of quality control
  - Policy issues. What needs to be implemented? What are the main barriers? Who are the key stakeholders?

d) Molecular alternatives

a. Presentation and discussion of alternative screening techniques: molecular testing
Presentation of information on molecular alternatives from the research team to the working group.

b. Flowchart: molecular alternatives
- What is the current screening implementation process here in Iquitos? How is it done? *The objective is to review each technique and its implementation process in detail.*
- What are the main issues related to these techniques? What are their advantages and disadvantages? *Variables to consider (the goal is to understand these and other influencing factors)*
  - Sensitivity (the effectiveness of the technique (lost cases, false positives, false negatives)
  - Cost (laboratory equipment, human resources, training, etc). How convenient/affordable is it in the Iquitos context?
  - Time (immediacy/delay of results)

- Laboratory needs (infrastructure, equipment)
- Logistical needs (e.g. does it require electricity? is sample transportation fast and effective?)
- High medical knowledge requirement (How demanding is it to implement? What type of training does it require? For whom? By whom?)
- Risks of overtreatment or undertreatment
- How equitable is it? Would the general population have access? How easy or difficult would it be?
- Feasibility of quality control
- Policy issues. What needs to be implemented? What are the main barriers? Who are the key stakeholders?

e) Voting process: comparing techniques

- Contrast: Having discussed the advantages and disadvantages of the techniques, compare them.
  c. Which would be the best technique for the Iquitos context? Why would it be the best option?
  d. Which technique would be completely ruled out as an option? Why?
- Combinations:
  e. What would be the best order for their implementation? Why?
  f. What are the benefits? What are the challenges?
- Challenges:
  g. What are the main barriers limiting the implementation of the techniques? (equipment needs, training needs, mentoring support, follow-up time, potential loss to follow-up)
  h. Are there logistical or policy barriers that obstruct the implementation of any screening technique?
  i. What barriers (if any) are present here in the Loreto region?
  j. Who are the key stakeholders in these issues?
- Strategies:
  k. What could be done to reduce these barriers? What (if anything) could make screening easier to implement here in Iquitos?
  l. What strategies can be implemented to address these challenges? What role could you play in these strategies?

f) Steps needed to improve the implementation

m. At the policy level
  - What is needed to improve the implementation of these techniques? What are the main challenges? Who are the key stakeholders?

n. Health professionals: Competence and training
  - How do you determine that you have conducted effective screening?
  - What objective and subjective measures do you use to evaluate your own screenings and those of others?
  - What are your ideas about quality indicators for the different techniques? What is important to you? How can quality control be incorporated into practice?
  - How could a feedback system for healthcare providers be implemented? Would it be useful? Why or why not?
  - How could your practice be improved?
  - What additional training could we provide to help? What would help you feel that you are providing the best possible care?

o. Laboratory

- What is needed in terms of laboratory? (infrastructure, equipment)

p. Cervical cancer screening quality service indicators

- What do you think is important for your patients?
- Do you think your patients are satisfied with the service provided? Why?
- How would you evaluate the current cervical cancer screening service indicators?
- How could they improve?

Variables to consider

- Patient-health professional interaction (warmth, willingness to answer questions, ways of addressing the patient, explanation of the procedure)
- Information on cervical cancer prevention and related ideas (¿information offered clearly in simple language? ¿use of illustrative materials?)
- Cleanliness of the environment, equipment and procedure
- Privacy for the conversation and the examination / interruptions
- Comfort with male and female staff. If the healthcare professional was male, was there a female assistant present?

q. Follow-up and results process

- How are abnormal results managed? What are the main challenges in handling this situation?
- What are your perceptions of the follow-up care for women with abnormal results? Are you satisfied with the care and referral systems? How can they improve?
- Are women being lost in the system? How do you manage your patients to reduce the chances of losing them in the system?
- Is it possible to create a follow-up registry? What would be the advantages? What difficulties might arise?
